# Enhanced recovery after bariatric surgery: an Italian consensus statement

**DOI:** 10.1007/s00464-022-09498-y

**Published:** 2022-08-11

**Authors:** Giuseppe Marinari, Mirto Foletto, Carlo Nagliati, Giuseppe Navarra, Vincenzo Borrelli, Vincenzo Bruni, Giovanni Fantola, Roberto Moroni, Luigi Tritapepe, Roberta Monzani, Daniela Sanna, Michele Carron, Rita Cataldo

**Affiliations:** 1 Bariatric Surgery Unit, IRCCS Humanitas Research Hospital, Rozzano, Milan Italy; 2 Bariatric Surgery Unit, Azienda Ospedale Università Padova, Padua, Italy; 3 Department of Surgery, San Giovanni di Dio Hospital, Gorizia, Italy; 4 Department of Human Pathology, University of Messina, Messina, Italy; 5 Bariatric Surgery Unit, Humanitas Gavazzeni, Bergamo, Italy; 6 Bariatric Surgery Unit, Campus Bio Medico University of Rome, Rome, Italy; 7 Bariatric Surgery Unit, ARNAS, G. Brotzu Hospital, Cagliari, Italy; 8 Bariatric Surgery Unit, Policlinico Sassarese, Sassari, Italy; 9 Department of Anesthesia and Intensive Care, San Camillo-Forlanini Hospital, Sapienza University of Rome, Rome, Italy; 10 Department of Anesthesia and Intensive Care Units, Humanitas Research Hospital, Humanitas University Milan, Rozzano, Milan, Italy; 11 Emergency Department, Section of Anesthesiology and Intensive Care, ARNAS, G. Brotzu Hospital, Cagliari, Italy; 12 Department of Medicine-DIMED, Section of Anesthesiology and Intensive Care, University of Padua, Via V. Gallucci, 13, 35121 Padua, Italy; 13 Unit of Anesthesia, Intensive Care and Pain Management, Campus Bio Medico University of Rome, Rome, Italy

**Keywords:** Obesity, Bariatric Surgery, Anesthesia, Enhanced recovery after surgery

## Abstract

**Background:**

Enhanced recovery after bariatric surgery (ERABS) is an approach developed to improve outcomes in obese surgical patients. Unfortunately, it is not evenly implemented in Italy. The Italian Society for the Surgery of Obesity and Metabolic Diseases and the Italian Society of Anesthesia, Analgesia, Resuscitation and Intensive Care joined in drafting an official statement on ERABS.

**Methods:**

To assess the effectiveness and safety of ERABS and to develop evidence-based recommendations with regard to pre-, intra-, and post-operative care for obese patients undergoing ERABS, a 13-member expert task force of surgeons and anesthesiologists from Italian certified IFSO center of excellence in bariatric surgery was established and a review of English-language papers conducted. Oxford 2011 Levels of Evidence and U.S. Preventive Services Task Force Grade Definitions were used to grade the level of evidence and the strength of recommendations, respectively. The supporting evidence and recommendations were reviewed and discussed by the entire group at meetings to achieve a final consensus.

**Results:**

Compared to the conventional approach, ERABS reduces the length of hospital stay and does not heighten the risk of major post-operative complications, re-operations, and hospital re-admissions, nor does it increase the overall surgical costs. A total of 25 recommendations were proposed, covering pre-operative evaluation and care (7 items), intra-operative management (1 item, 11 sub-items), and post-operative care and discharge (6 items).

**Conclusions:**

ERABS is an effective and safe approach. The recommendations allow the proper management of obese patients undergoing ERABS for a better outcome.

Bariatric surgery is the most effective treatment for morbid obesity. The demand for bariatric surgery continues to grow as the proportion of the population with obesity increases. Bariatric surgery is mainly optional in predominantly high-risk patients. Providing an effective, safe, and economically sustainable approach is challenging for physicians [1, 2].

Enhanced Recovery after Surgery (ERAS) is a modern approach to reduce perioperative stress and help patients recover more quickly following surgery, leading to improved outcomes in postoperative morbidity and length of hospital stay (LOS). ERAS, implemented initially for elective colorectal surgery, has been extended over time to many other surgeries [3], including bariatric surgery [4]. However, the ERAS after bariatric surgery (ERABS) guidelines published in 2016 [4] relied heavily on the evidence and recommendations developed in no-bariatric surgery settings. Afterward, an increasing number of peer-reviewed papers about ERABS have been published, showing the favorable outcomes observed in ERAS.

Despite the evidence, some resistance to adopting ERABS protocols remains in bariatric surgery centers in Italy. The Italian Ministry of Health’s 2018 Annual Report on hospital admission and discharge events reported a total of 25,424 surgical procedures for obesity, with an average LOS of 3.9 days [5]. So, if, on the one hand, this data suggests that the conventional approach is largely adopted in bariatric surgery, on the other, it leaves considerable opportunity for improving bariatric surgery outcomes by adopting the ERABS approach.

## Materials and methods

The Italian Society of Surgery of Obesity and Metabolic Diseases (SICOB) and the Italian Society of Anesthesia, Analgesia, Resuscitation and Intensive Care (SIAARTI) undertook a process for drafting an official joint statement on ERABS. The two societies appointed a 13-member expert task force, which met first in October 2019 to define the scope and methods of the project. It was decided that the primary objective was to summarize available evidence supporting ERABS, focusing on the efficacy and safety outcomes. The secondary objective was to review the importance of the individual ERABS protocol items.

Four main areas for investigation were identified: outcomes for the primary endpoint and preoperative, intraoperative, and postoperative care for the secondary endpoint. Corresponding subcommittees were appointed to systematically review ERABS topics, grade the levels of evidence, and propose specific recommendations with supporting evidence for each topic.

A systematic PubMed search of English-language papers published from 1999 to April 2020 was performed combining the following terms: “obesity” and “surgery” or “bariatric surgery” or “fast track” or “enhanced recovery” or “perioperative care” or “perioperative management” or “sleeve gastrectomy” or “gastric bypass.” The levels of evidence were assessed using Oxford 2011 Levels of Evidence (Table 1) [6]. To rate the strength of recommendations, grade definitions based on the U.S. Preventive Services Task Force were used (Tables 2 and 3) [7].

**Table 1:** Grading of level of evidence (from Oxford Centre for Evidence-Based Medicine 2011 Level of Evidence) [6]

Question	Step 1 (Level 1*)	Step 2 (Level 2*)	Step 3 (Level 3*)	Step 4 (Level 4*)	Step 5 (Level 5*)
How common is the problem?	Local and current random sample surveys (or censuses)	Systematic review of surveys that allow matching to local circumstances**	Local non-random sample**	Case-series**	n/a
Is this diagnostic or monitoring test accurate? (Diagnosis)	Systematic review of cross sectional studies with consistently applied reference standard and blinding	Individual cross sectional studies with consistently applied reference standard and blinding	Non-consecutive studies, or studies without consistently applied reference standards**	Case–control studies, or “poor or non-independent reference standard**	Mechanism-based reasoning
What will happen if we do not add a therapy? (Prognosis)	Systematic review of inception cohort studies	Inception cohort studies	Cohort study or control arm of randomized trial*	Case-series or casecontrol studies, or poor quality prognostic cohort study**	n/a
Does this intervention help? (Treatment Benefits)	Systematic review of randomized trials or n-of-1 trials	Randomized trial or observational study with dramatic effect	Non-randomized controlled cohort/follow-up study**	Case-series, case–control studies, or historically controlled studies**	Mechanism-based reasoning
What are the COMMON harms? (Treatment Harms)	Systematic review of randomized trials, systematic review of nested case–control studies, n of-1 trial with the patient you are raising the question about, or observational study with dramatic effect	Individual randomized trial or (exceptionally) observational study with dramatic effect	Non-randomized controlled cohort/follow-up study (post-marketing surveillance) provided there are sufficient numbers to rule out a common harm. (For long-term harms the duration of follow-up must be sufficient.)**	Case-series, case–control, or historically controlled studies**	Mechanism-based reasoning
What are the RARE harms? (Treatment Harms)	Systematic review of randomized trials or n-of-1 trial	Randomized trial or (exceptionally) observational study with dramatic effect			
Is this (early detection) test worthwhile? (Screening)	Systematic review of randomized trials	Randomized trial	Non-randomized controlled cohort/follow-up study**	Case-series, case–control, or historically controlled studies**	Mechanism-based reasoning

*Level may be graded down on the basis of study quality, imprecision, indirectness (study PICO does not match questions PICO), because of inconsistency between studies, or because the absolute effect size is very small; Level may be graded up if there is a large or very large effect size

**As always, a systematic review is generally better than an individual study

**Table 2:** Grading of quality of evidence (from US Preventive Services Task Force) [7]

Grade	Description
A	The available evidence usually includes consistent results from multitude of well-designed, well-conducted, studies in representative care populations. These studies assess the effects of the service on the desired health outcomes. Because of the precision of findings, this conclusion is, therefore, unlikely to be strongly affected by the results of future studies. These recommendations are often based on direct evidence from clinical trials of screening, treatment, or behavioral interventions. High-quality trials designed as “pragmatic” or “effectiveness” trials are often of greater value in understanding external validity
B	The available evidence is sufficient to determine the effects of the service on targeted health outcomes, but confidence in the estimate is constrained by factors such as the number, size, or quality of individual studies in the evidence pool; some heterogeneity of outcome findings or intervention models across the body of studies; mild-to-moderate limitations in the generalizability of findings to routine care practice. As more information becomes available, the magnitude or direction of the observed effect could change, and this change may be large enough to alter the conclusion
C	The available evidence is insufficient to assess effects on health outcomes. Evidence is insufficient because of the very limited number or size of studies Inconsistency of direction or magnitude of findings across the body of evidence; critical gaps in the chain of evidence; findings are not generalizable to routine care practice; a lack of information on prespecified health outcomes; lack of coherence across the linkages in the chain of evidence. More information may allow an estimation of effects on health outcomes

**Table 3:** Grading of the strength of recommendation (from U.S. Preventive Services Task Force) [7]

Grade	Definition	Suggestion for practice
A	The USPSTF recommends the service. There is high certainty that the net benefit is substantial	Offer or provide this service
B	The USPSTF recommends the service. There is high certainty that the net benefit is moderate or there is moderate certainty that the net benefit is moderate to substantial	Offer or provide this service
C	The USPSTF recommends selectively offering or providing this service to individual patients based on professional judgment and patient preferences. These is at least moderate certainty that the net benefit is small	Offer or provide this service for selected patients depending on individual circumstances
D	The USPSTF recommends against the service. There is moderate or high certainty that the service has no net benefit or that the harms outweigh the benefits	Discourage the use of this service
I	The USPSTF concludes that the current evidence is insufficient to assess the balance of benefits and harms of the service. Evidence is lacking, of poor quality, or conflicting, and the balance of benefits and harms cannot be determined	Read the clinical considerations section of USPSTF Recommendation Statement. If the service is offered, patients should understand the uncertainty about the balance of benefits and harms

Key issues were discussed at a meeting in January 2020, after which a comprehensive document was circulated and then revised. The panel concluded its work with a Consensus Meeting in July 2020, where supporting evidence and recommendations were reviewed and discussed by the entire group to achieve a final consensus. Subsequently, a draft report was prepared and sent to the panel for comments and modification. Each author approved the final version before it was submitted. Ethics approval and consent were not required for this type of study.

## Results

The evidence and recommendations concerning the primary safety and efficiency endpoints are summarized in Table 4. A total of 25 recommendations were proposed, covering pre-operative evaluation and care (7 items), intra-operative management (1 item, 11 sub-items), and post-operative care and discharge (6 items). The levels of evidence and recommendations for each item in the ERABS protocol are summarized in Table 4.

**Table 4:** Effectiveness, safety, and items of Enhanced Recovery after Bariatric Surgery (ERABS) compared to standard approach

	Evidence		Strength of	Expert task force statement
	Level	Quality	Recommendation	
*Effectiveness and safety of ERABS*				
Length of hospital stay	1	A	A	ERABS reduces the duration of hospital stays
Risk of complications	1	A	A	ERABS is a safe approach for obese patients
Cost of surgery	2	B	A	Adopting an ERABS protocol does not increase the cost of surgery
*Items ERABS. Preoperative care*				
Information and counseling	2	B	A	The information provided to the patient should not be limited to what is required for informed consent for both surgery and anesthesia; it should be adequate to provide realistic expectations of the ERABS approach
Patient optimization	1	A	A	Pre-operative optimization through smoking cessation, weight loss, blood glucose control, and the use of non-invasive ventilation (when indicated) is recommended in ERABS
Fasting	1	A	A	Clear liquids and solid food are recommended up to 2 h and 6 h, respectively, prior to the induction of anesthesia in ERABS
PONV prophylaxis	1	A	A	Strategies aimed at minimizing the risk of post-operative nausea and vomiting after general anesthesia are recommended for better patient outcomes in ERABS
Venous thromboembolism prophylaxis	2	B	A	Multimodal venous thromboembolism prophylaxis, including early patient mobilization, is recommended in ERABS
Antibiotic prophylaxis	2	B	A	Pre-operative intravenous antibiotic prophylaxis is recommended in ERABS
Monitoring	1	A	A	Proper perioperative monitoring is recommended in ERABS
*Items ERABS. Intraoperative care*				
Standardized anesthesia protocol	1	A	A	A standardized anesthesia approach is recommended in order to optimize outcomes in ERABS
1-Airways management	1	A	A	A careful airways assessment is recommended in ERABS
2-Preoxygenation	1	A	A	An adequate preoxygenation performed in ramped position is recommended in ERABS
3-Tracheal intubation	1	A	A	Proper airway management in order to minimize difficulties is recommended in ERABS
4-General anesthesia	1	A	A	General anesthesia is the anesthesiologic approach of choice in ERABS
5-Neuromuscular blockade	1	A	A	Proper neuromuscular blockade management is recommended in ERABS
6-Analgesia opioid sparing	1	A	A	Opioid-sparing or opioid-free anesthesia is recommended in ERABS
7-Multimodal analgesia	1	A	A	Multimodal analgesia is recommended in ERABS to optimize pain control after surgery and to reduce or eliminate the use of opioids in the post-operative period
8-Locoregional anesthesia	1	A	A	Locoregional anesthesia supports and complements general anesthesia in ERABS
9-Protective lung ventilation	1	A	A	Protective mechanical lung ventilation during general anesthesia is recommended in ERABS
10-Goal-directed fluid therapy	3	B	A	Proper perioperative fluid management is recommended. Goal-directed fluid therapy should be considered in ERABS
11-Protected extubation	1	A	A	Extubation should be performed on an awake patient in the ramped position in ERABS
*Items ERABS. Postoperative care*				
Nasogastric tube	1	A	A	Routine placement of the SNG does not improve outcomes in ERABS
Abdominal drainage	2	B	A	Routine use of abdominal drainage should be discontinued in ERABS
Bladder catheter	4	C	A	Routine use of bladder catheters should be abandoned in ERABS
Early mobilization	3	B	A	Early post-operative mobilization is recommended in ERABS
Early re-feeding	1	A	A	Early post-operative resumption of oral feeding is recommended in ERABS
Early discharge	1	A	A	Early discharge of the patient is recommended in ERABS. Adoption and verification of a discharge checklist upon discharge are recommended in ERABS

### Effectiveness and safety of ERABS

Compared to the conventional approach, ERABS reduces the LOS [8–10], independently of the type of surgical procedure performed [11, 12]. The more successful ERABS items the multidisciplinary team adopts, the greater the likelihood of a reduced post-operative LOS [13].

Also, compared to the standard approach, ERABS does not have an increased risk for major postoperative complications, reoperations, and hospital readmissions [8–12, 14]. However, it also does not reduce the number of major complications [8–12, 14]. ERABS, moreover, seems to reduce the total surgical costs compared to the standard approach [8, 15].

### Preoperative counseling

Preoperative information and counseling are key items for managing the expectations of patients and preparing them for early discharge [16]. Counseling was one of the most frequently used items in a retrospective multicenter study [17] and one of the key items in 11 of the 13 studies considered in a systematic review [8]. A retrospective study on the safety of an early postoperative discharge following bariatric surgery identified the receipt of preoperative information on early ambulation and refeeding, as well as pain and postoperative nausea and vomiting (PONV) management, as one of the more important protocol items of the ERABS pathway [18].

### Preoperative patient optimization

Smoking cessation for at least four weeks reduced postoperative surgical and pulmonary complications by 41% [19], supporting the argument that smoking should be discontinued for at least four weeks before bariatric surgery [20]. Physicians should inform patients of the increased risk of morbidity and mortality in smokers. This risk decreases the longer smoking is ceased before surgery [21].

Preoperative weight loss reduces liver volume and may technically facilitate the operation [22]. However, whether weight loss reduces postoperative complications remains controversial [21, 23]. A Swedish registry study reported a decrease in complications after gastric bypass surgery [24].

Optimizing the preoperative fasting blood glucose level through diet, physical activity, and pharmacotherapy is mandatory [20, 21, 23]. A value greater than 180 mg/dl was associated with increased perioperative complications and mortality [20].

Obstructive sleep apnea (OSA) carries an increased risk of postoperative cardiorespiratory complications [25]. Although this aspect has been debated in bariatric surgery [26, 27], increased complications and LOS have been observed in OSA patients who underwent bariatric surgery [28–30]. The use of noninvasive ventilation (NIV) (e.g., continuous positive airway pressure [CPAP]) was reported to improve the preoperative cardiometabolic profile [31] and the postoperative respiratory function [21, 23, 32]. It may be safely adopted for patients undergoing bariatric surgery [32]. Even if postoperative care in a monitored setting may be considered for high-risk obese patients undergoing surgery [20, 33], patients with severe OSA and/or home CPAP selected for ERABS do not require a routine planned postoperative admission into the intensive care unit [20, 21, 27].

### Preoperative fasting

Properly managing preoperative fasting may be important to minimize perioperative stress. Maintaining homeostasis avoids or reduces catabolism and related proteolysis, asthenia, or cellular dysfunction [34–37]. Clear liquids can be taken up to 2 h before surgery and solid food up to 6 h before the induction of anesthesia [3, 38–41]. No strong evidence supports preoperative oral carbohydrate loading in bariatric surgery. Furthermore, there is some reluctance to adopt preoperative oral carbohydrate loading in patients suffering from diabetes or metabolic syndrome [42]. Absolute or prolonged preoperative fasting is no guarantee of a secretion-free stomach at the induction of anesthesia [42].

### Postoperative nausea and vomiting prophylaxis

General anesthesia is associated with an increased risk of PONV [43]. The prevention of PONV in ERABS is recommended [4], and it is consistent with the guidelines for the general surgical population [43]. The polypharmacological approach to PONV prophylaxis is preferable to monotherapy [20, 43] and was reported to decrease the incidence of PONV and the postoperative use of antiemetics, opioid analgesics, and liquid infusion [44].

### Venous thromboembolism prophylaxis

The incidence of deep vein thrombosis and pulmonary embolism after bariatric surgery is approximately 0.1–0.5% and, in the vast majority of cases, occurs after the patient is discharged [21, 45, 46]. A multimodal venous thromboembolism prophylaxis via chemoprophylaxis, mechanical aids, and/or patient mobilization is suggested [4, 20, 21, 47–49]. It is reported as a key item in ERABS [12, 34, 39, 50–61]. Chemoprophylaxis with low-molecular-weight heparin (LMWH) after surgery and home discharge is considered effective [20, 48, 52, 53] and is associated with a lower bleeding risk [52]. However, there is insufficient evidence to recommend a specific dose and duration of the LMWH treatment [20, 47–49, 52]. Early ambulation in the postoperative period is considered a useful component of multimodal venous thromboembolism prophylaxis [20, 47, 49].

### Antibiotic prophylaxis

The incidence of surgical site infections in obese patients varies from 1 to 21.7%, depending on the procedure type [62]. The use of preoperative antibiotic prophylaxis is then recommended [20, 21, 23]. It should follow the standard guidelines for perioperative antibiotic prophylaxis [63]. The most frequently used antibiotic is cefazolin (dosage 1–4 g), with clindamycin recommended as an alternative in allergic patients [54, 64]. A dose adjustment based on the patient’s weight compared to a fixed dose of 2 g administered intravenously before surgical incision is preferable [65, 66]. Higher dosages (cefazolin 3 g) in patients weighing > 120 kg should be considered [20]. Literature does not support prophylactic vancomycin or cefoxitin [64]. Intestinal preparation by antibiotic prophylaxis (whether or not combined with mechanical preparation) is not recommended in bariatric surgery [60]. Preoperative antibiotic prophylaxis, administered intravenously at the induction of anesthesia or 30–60 min before surgical incision, is reported to be one of the important items in ERABS [12, 17, 60, 67].

### Monitoring

The standard for anesthesia monitoring should be ensured in the perioperative period [4, 20, 33, 68]. In obese patients, anesthesia depth monitoring ensures a more accurate induction of anesthesia with propofol and reduces the risk of awareness from inadequate dosing [69], as well as during intravenous and inhalational anesthesia [70]. The monitoring of the neuromuscular function helps reduce the risk of postoperative respiratory complications [71]. Temperature monitoring reduces the risk of hypothermia, improving postoperative recovery [72].

### Standardized anesthesia approach

The standardized ERABS approach showed favorable outcomes compared to a non-standardized one [8–14]. Advances in the perioperative care of obese patients translated into a standardized anesthesiological approach have been proven to be effective and safe [73] in the ERABS context as well [39].

### Airway management

In obese surgical patients, airway management can present challenges. Difficult mask ventilation has been reported in 8.8% of obese patients, and 11% of those have morbid obesity. Difficult intubation has been reported in 3.3–16.7% of patients [20]. Difficult airway management predictors include males, OSA, increased waist-to-hip ratio (> 0.8 in women and > 0.9 in men), BMI (> 50 kg/m^2^), and neck circumference (> 41 cm in women and > 43 cm in men) [20, 33, 74–76]. OSA risk stratification is then suggested using validated questionnaires, such as STOP-BANG, and reserving the time-consuming and expensive polysomnography for patients at high risk of severe OSA (e.g., STOP-BANG ≥ 5) [3, 21, 23, 28–31, 33, 77–81]. The ramp position improves the likelihood of successful airway management in obese patients [20, 23].

### Pre-oxygenation

Adequate pre-oxygenation aiming for end-tidal oxygen concentrations of ≥ 90% before the induction of general anesthesia is suggested. Pre-oxygenation has been reported as important in prolonging safe apnea times following general anesthesia induction [82, 83]. The NO DESAT (nasal oxygen during efforts securing a tube) technique, which uses a simple nasal cannula with standard cold dry oxygen may be considered [82]. Pre-oxygenation using a high-flow nasal cannula or positive pressure by CPAP/NIV seems more effective than the standard approach [82, 84–91] and may be beneficial for high-risk obese patients [20, 32, 33, 82].

### Tracheal intubation

An appropriate planned approach is recommended for airway management in obese patients [3, 20, 33]. In obese patients, videolaryngoscopes compared with Macintosh laryngoscopes increases the likelihood of successful intubation on the first attempt at laryngoscopy [92]. This result seems to be ensured more by the use of a videolaryngoscope with a tracheal tube guide than one without [93].

Second-generation extraglottic devices were recommended as rescue devices for pulmonary oxygenation/ventilation in the case of difficult airway management and possible fibroscope-guided intubation [94–98]. The fibroscopic/endoscopic technique was essential when intubating conscious patients [99]. In awake patient, video laryngoscopy has been suggested as a valid alternative to fiberoptic intubation in experienced practitioners [100–102].

### General anesthesia

General anesthesia is the approach of choice in ERABS [3, 4, 20, 33]. There is no evidence supporting the superiority of inhalation versus intravenous anesthesia [3, 4, 20, 33]. Anesthesia strategies based on short-acting, low-accumulation drugs that promote rapid recovery from general anesthesia are suggested [4, 20, 33]. Desflurane has been associated with faster postoperative awakening and recovery than other inhalational anesthetics and propofol [103–106]. Intravenous anesthesia with propofol has demonstrated a lower risk of PONV in the general population [107] and in obese surgical patients [108]. PONV prophylaxis has been observed to reduce the risk of PONV incidence [109], particularly with inhalational anesthesia [20].

### Analgesia and opioid

In the general surgical population, opioid use was associated with an increased risk of PONV [110], as well as upper airway obstruction and hypoventilation [111]. In obese patients, intraoperative opioid use was associated with an increased risk of PONV [112] and postoperative respiratory complications [113]. *Opioid-sparing* or *opioid-free* anesthesia should be preferred when managing obese surgical patients [20, 33] under ERABS [4], as it is associated with a lower incidence of PONV [112].

Opioids with rapid elimination kinetics, such as remifentanil, have demonstrated faster postoperative awakening and recovery of respiratory functions in general surgical populations compared to other opiods [114]. In obese patients, remifentanil has demonstrated reduced recovery time from general anesthesia, respiratory complications, and LOS [115]. Patient-controlled analgesia has also been successfully used in obese surgical patients [116] and should be preferred over continuous infusion in the postoperative period [3, 20].

### Multimodal analgesia

Multimodal analgesia as a pain control strategy using analgesics (e.g., nonsteroidal anti-inflammatory drugs, paracetamol) in combination with analgesic adjuvants and local anesthetics is recommended in ERABS to optimize pain control after surgery and to reduce or eliminate the postoperative use of opioids [4, 20, 33, 34, 117] and related adverse events [118]. Adjuvant analgesics, such as ketamine [119, 120], clonidine [119, 121], dexmedetomidine [121–123], magnesium [123–125], lidocaine [126], pregabalin [127, 128], and gabapentin [129], alone or in combination, significantly reduced postoperative pain compared with controls.

### Locoregional analgesia

Intraperitoneal local anesthetics [130–133] and the ultrasound-guided TAP (transversus abdominis plane) block [134–136] showed a significant effect on reducing pain scores at recovery from general anesthesia [137, 138]. The benefit may be increased by the infiltration of trocar insertions and wound closures with local anesthetics at the end of the surgical procedure [20, 135]. Notably, these pain control benefits did not affect the LOS [130–136]. Epidural analgesia may be considered in selected patients [139].

### Neuromuscular blockade

Neuromuscular blockade (NMB) is suggested at the induction of general anesthesia to facilitate airway management and subsequent pulmonary ventilation [20, 33, 82]. Deep compared to moderate NMB optimizes the surgical field view, reduces procedural complications in laparoscopic surgery [140], and is associated with less postoperative pain [140, 141]. Sugammadex compared to cholinesterase inhibitors provides a more rapid and predictable recovery of rocuronium-induced NMB [142, 143] and is associated with less pain and PONV in the postoperative period [141, 144] and faster discharge to the surgical ward [143, 144]. Quantitative monitoring and complete recovery of the neuromuscular function at the end of surgery are highly recommended [4, 20, 33].

### Protective ventilation

Protective mechanical lung ventilation should be preferred for obese patients undergoing general anesthesia for bariatric surgery [20] because it is associated with reduced postoperative respiratory complications, LOS, and mortality in both non-obese and obese surgical patients [145–147].

Slow abdominal insufflation with a maximum intra-abdominal pressure of less than 15 mmHg is advised, when possible, during laparoscopy [20] to favor mechanical lung ventilation [20, 148]. This strategy, combined with limiting surgical time, is associated with a reduction in the risk of postoperative respiratory complications [148–150].

### Goal-directed fluid therapy

Proper perioperative fluid management avoiding overhydration helps to minimize the risk of PONV, postoperative complications, and prolonged LOS [151–153]. A goal-directed fluid therapy (GDFT) has been suggested as an adequate strategy to reduce these risks [154], even in bariatric surgery [155–157]. In bariatric surgery, excessive fluid should be avoided, and GDFT should be considered a useful strategy [155–157]. A GDFT guided by noninvasive indices, such as the Pleth Variability Index, may be a more acceptable monitoring option to GDFT based on invasive methods [155] and, consequently, more widely used in bariatric surgery, even in the ERABS context [155–157]. Postoperative fluid infusions should be discontinued as soon as possible, with preference given to the enteral route [60, 153, 158]. Intraoperative hypotension (mean arterial pressure of ≤ 65 mmHg), even for a few minutes, is a predictor of renal and myocardial damage [159]. It should thus be avoided or promptly treated in the perioperative period [154, 159].

### Protected extubation

The extubation should be performed on an awake patient in the ramped position and/or reverse Trendelenburg position, which improves lung volume, oxygenation, and respiratory mechanics [20, 33, 160, 161]. Oxygen therapy with nasal goggles or HFNC was associated with a reduced risk of post-extubation desaturation [160] and reintubation [162]. CPAP or NIV was recommended for awakening moderate-severe OSA patients or those suffering from the obese hypoventilation syndrome who are already receiving home treatment or who will require opioid therapy postoperatively [20, 32, 163]. CPAP and NIV do not appear to negatively affect the outcome of the surgical procedure [32].

### Nasogastric tube

In abdominal surgery, avoiding the routine use of nasogastric tubes (NGTs) results in a faster recovery of the bowel function, a decrease in pulmonary complications, and a shorter LOS without any associated increase in anastomotic dehiscence [164]. In ERABS, compared to the conventional approach, avoiding NGT use demonstrated better postoperative recovery without an increase in complications [11–13, 41], reducing postoperative pain and PONV, promoting early mobilization and resumption of liquid diet, and resulting in better compliance at discharge [38, 39, 55]. Society positions in favor of abandoning, when possible, the routine use of postoperative NGT are already available [3, 54].

### Abdominal drainage

Many gastrointestinal surgeries can be performed safely without prophylactic drainage [165]. In bariatric surgery, the evidence is limited [166]. A retrospective study of a gastric bypass population reported no difference in anastomotic dehiscence and reintervention rates in patients receiving abdominal drainage compared to those who are not [167]. Avoiding abdominal drainage was found to be a key item in the ERABS [3, 11, 12, 14, 17, 19, 59, 67, 168]. Society positions in favor of abandoning the routine use of abdominal drainage, when possible, are available [3, 54].

### Bladder catheter

Avoidance or early removal of bladder catheters resulted in early mobilization and prevented urinary tract infection [169–171]. In ERABS, compared to the conventional approach, avoiding bladder catheters was associated with early mobilization and reduced LOS, readmission rate, and minor complications, such as urinary tract infection [3, 10, 14, 18, 50, 67, 168, 172]. Society positions in favor of abandoning the routine use of bladder catheter, when possible, are available [54].

### Early mobilization

Early postoperative mobilization is recommended in obese patients undergoing bariatric surgery [4, 11, 14, 20, 21, 33]. Independent mobilization for at least 4 h during the first 24 h following surgery was significantly associated with a lower rate of postoperative complications and a shorter LOS [173]. Early postoperative mobilization was reported as a key element of better postoperative recovery in ERABS [17–20, 29, 39, 54–59, 67, 168, 172], and adequate independent mobilization was a condition for a home discharge [14, 18, 21, 39, 54, 56, 58, 59, 67].

### Early refeeding

The advantage of early oral refeeding has been reported in both ERAS [3] and ERABS [39–41, 173] and seems particularly associated with an earlier restoration of bowel function, faster wound healing, less infection, and lower risk of postoperative complications [36, 37, 174]. Early oral refeeding was associated with a reduced LOS and mortality [44, 60]. Conversely, prolonged postoperative fasting was associated with thirst, emotional fixation on food, and a phobia about the reintroduction of food [36, 37, 174].

### Discharge

Discharge on postoperative day 1 or 2 was not associated with an increase in the complication rate, readmission, or telephone consultations in both ERAS [3, 18] and ERABS [11, 12, 175], particularly in the absence of significant comorbidities [176, 177]. Caution should be taken in the presence of suspected clinical conditions (e.g., tachycardia) and/or abnormal level of serological markers (e.g., C-reactive protein, procalcitonin) as predictors of risk of postoperative complications [178–181]. However, there is no consensus on which serological markers should be assessed and at which postoperative time point [182–185]. Routine postoperative contrast imaging examination increased costs and LOS [186, 187] and was not considered to be a reliable assessment of postoperative complications [188–190].

Discharge on the day of surgery was reported as feasible in selected patients [190–192]. However, there was some disagreement around the safety of same-day discharge [193, 194]. The adoption and verification of a discharge checklist may be a useful tool for a safe discharge [57]. The minimum criteria for a discharge included vital parameters within the normal range, adequate pain control by nonopioid analgesics, adequate water intake, ability to tolerate liquid diet, and no evidence of sepsis or signs of postoperative complication. Finally, predischarge education concerning signs and symptoms of possible postoperative complications and procedures for contacting the staff were also identified as a key component of a successful discharge [11, 12, 39, 55].

## Discussion

Literature review supports ERABS in reducing LOS without increasing complications and costs [8–10]. Producing a statement on ERABS standardizes the ERABS approach across bariatric surgery centers, benefiting both patients and hospitals [195]. The standardization of the approach may also allow the evaluation of ERABS’s effective impact on the postoperative complications and total costs compared to the standard approach [196]. Notably, it became clear from this statement that the more items in the protocol that are adopted, the more efficient the ERABS approach will be [13].

Perioperative management involves a multidisciplinary team, where a full collaboration among anesthesiologists, surgeons, and staff members may lead to the best results [73]. The ERABS statement, drafted thanks to this collaboration, is, therefore, addressed not only to anesthesiologists or surgeons, but also to everyone involved in the perioperative care of obese patients in bariatric surgery centers.
